# Imputing missing values in single-cell RNA-sequencing data: a statistical and machine learning-based approach

**DOI:** 10.1093/bib/bbag072

**Published:** 2026-02-16

**Authors:** A F M Shamsuzzaman, Sumanta Ray, Anirban Mukhopadhyay

**Affiliations:** Department of Computer Science, Raja Rammohun Roy Mahavidyalaya, Radhanagar, Nangulpara, Hooghly, West Bengal 712406, India; Data Science, The West Bengal National University of Juridical Sciences, Salt Lake City, Kolkata, West Bengal 700106, India; Department of Computer Science and Engineering, University of Kalyani, Kalyani, Nadia, West Bengal 741235, India

**Keywords:** single-cell RNA-sequencing, dropout, imputation, regression, clustering, downstream analysis

## Abstract

Single-cell RNA sequencing (scRNA-seq) offers a powerful tool to capture gene expression patterns within individual cells. However, due to the limited RNA content within cells, dropout events occur, resulting in a substantial number of zero counts in the single-cell expression matrix. To address this issue, we propose a novel method called single-cell dropout detection and imputation (scDDI). This method identifies dropout events using a Poisson–negative binomial mixture model and subsequently imputes the missing values using a decision tree regression model. We evaluate the performance of scDDI on both simulated and real scRNA-seq datasets, demonstrating its superiority over established single-cell imputation techniques. Notably, scDDI significantly improves dropout detection, leading to enhanced performance in various downstream analysis tasks like gene expression recovery, cell clustering, and cell subpopulation identification.

## Introduction

Single-cell RNA sequencing (scRNA-seq) technology enhances traditional gene expression profiling by enabling high-throughput transcriptomic analysis at the single-cell level [1]. However, a significant challenge in scRNA-seq is the sparsity of the gene expression matrix that contains numerous zero or near-zero values. These zeros arise primarily due to the dropout effect [2], where incomplete RNA capture results in undetected gene expression. Since many downstream analyses [3] in scRNA-seq rely on accurate gene expression estimates, imputing missing values is essential. This task is particularly challenging because zero-valued gene expression measurements arise from two distinct sources: (i) biological zeros, representing the genuine absence of gene expression in a cell at the time of sequencing, and (ii) technical zeros, caused by incomplete transcript capture, wherein a gene is expressed but remains undetected. Effective imputation approaches must, therefore, first discriminate between technical dropouts and true biological absence, and subsequently estimate the missing expression levels with high accuracy.

A wide range of computational methods [4, 5] have been developed for imputation, which can be broadly categorized into four main groups [6]: (i) data smoothing methods, (ii) model-based methods, (iii) low-rank matrix-based methods, and (iv) deep learning-based methods.

Data smoothing methods typically estimate missing gene expression values by leveraging information from similar cells. Examples of such methods include DrImpute [7], kNNImpute [8], MAGIC [9], PRIME [10], and scNPF [11]. DrImpute identifies clusters of similar cells and imputes dropout values by averaging the gene expression levels within these clusters. The Weighted k-Nearest Neighbors Imputation method (kNNImpute) selects genes with expression patterns similar to the gene affected by dropout to estimate the missing values. MAGIC applies the concept of thermal diffusion, imputing dropout values by constructing graphs that capture the relationships among similar cells. PRIME builds cell-type-specific subnetworks to estimate missing expression levels, thereby improving the separation of cell types and enhancing downstream visualization. scNPF integrates prior gene interaction networks with single-cell measurements to combine global topological structure with context-specific variation.

Model-based methods address imputation by explicitly modeling gene expression as a mixture of distributions corresponding to biological expression and dropout events. Notable examples include scImpute [12], SAVER [13], SAVER-X [14], scDoc [15], and BISCUIT [16]. scImpute employs a Gaussian distribution to model true gene expression and a Gamma distribution for dropout events, using the Expectation–Maximization (EM) [17] algorithm to estimate model parameters and impute missing values. SAVER adopts a Poisson–Gamma mixture with a Bayesian prior to borrow strength across genes and estimate true expression values, whereas SAVER-X extends this framework by incorporating information from a pretrained deep autoencoder to facilitate transfer learning across datasets. scDoc similarly employs a probabilistic mixture formulation to correct for dropout noise while preserving biological structure. BISCUIT adopts a hierarchical Bayesian mixture model with cell-specific scaling factors integrated into a hierarchical Dirichlet process [18].

Low-rank matrix-based methods recover gene expression by exploiting linear dependencies among cells and reconstructing the expression matrix from a low-rank approximation. Representative approaches include Adaptively thresholded Low-Rank Approximation (ALRA) [19], McImpute [20], single-cell Robust Matrix Decomposition (scRMD) [21], cell sub-population based bounded low-rank (PBLR) [22], and WEighted Decomposition of Gene Expression (WEDGE) [23]. ALRA applies singular value decomposition to generate a low-rank representation of the observed matrix, replacing entries below a predefined threshold with zeros. McImpute treats dropout recovery as a matrix completion problem using nuclear norm minimization [24] to separate true from technical zeros. scRMD performs robust matrix decomposition to impute missing entries while mitigating noise with minimal parametric assumptions. PBLR accounts for cellular heterogeneity by partitioning cells into subpopulations before applying bounded low-rank recovery within each group. WEDGE employs weighted matrix factorization to better preserve cell–cell and gene–gene correlations in highly sparse datasets.

Deep learning-based methods infer the potential spatial representation of cells using established deep learning techniques and then reconstruct the observed expression matrix from this estimated space. Examples of such methods include deep count autoencoder (DCA) [25], DeepImpute [26], scVI [27], scGNN [28], and scScope [29]. DCA extends the conventional autoencoder by integrating a noise model into its loss function to account for dropouts. DeepImpute is a neural network-based approach employing dropout layers and tailored loss functions to learn data patterns for accurate imputation. scVI employs stochastic optimization and deep neural networks to model gene expression distributions by aggregating information across similar cells and genes, while explicitly correcting for batch effects and limited sensitivity. scGNN formulates imputation as a graph learning task, propagating information across cellular neighborhoods via graph neural networks [30]. scScope adopts a recurrent denoising framework to reconstruct large-scale single-cell data and improve cell-type resolution.

Despite significant progress, scRNA-seq imputation methods differ in their underlying assumptions and behavior. Smoothing approaches are efficient but prone to oversmoothing; model-based methods rely on restrictive distributional assumptions; low-rank recovery captures global linear structure but not local nonlinear relationships; and deep learning frameworks require large datasets and lack interpretability. These limitations motivate hybrid approaches that integrate dropout modeling with mechanisms that preserve cellular heterogeneity and local transcriptional context.

In this article, we introduce single-cell RNA-seq dropout detection and imputation (scDDI), a hybrid framework built on three core steps: (i) estimating dropout probabilities with a PNB model and computing cell–cell similarity through a dropout-aware weighted cosine similarity; (ii) identifying the most relevant neighboring cells; and (iii) imputing missing values using a decision tree regressor that captures local expression patterns. What distinguishes scDDI from existing approaches is its combination of dropout-aware similarity learning, in which gene–cell-specific dropout probabilities directly influence neighborhood construction, and a local nonlinear imputation strategy that adapts to the transcriptional context of each cell. Instead of global probabilistic predictions or gene–gene network models, scDDI performs local, dropout-aware imputation using decision tree regression within dynamically selected neighborhoods. This enables flexible, nonlinear recovery with respect to each cell’s local transcriptional context. Together, these components form a coherent and nontrivial framework that handles sparsity more effectively than traditional smoothing, global models, or deep-learning-based imputation methods.

Across seven simulated and seven real scRNA-seq datasets, scDDI shows consistent gains in multiple aspects of performance. It improves expression recovery [Pearson and Spearman correlations, root mean square error (RMSE), and mean absolute error (MAE)], enhances clustering accuracy [adjusted rand index (ARI) [31] and normalized mutual information (NMI) [32]], and achieves strong precision, recall and F1-score in identifying dropout events. Visualizations via UMAP [33] embeddings further confirm that scDDI produces clearer subpopulation structure while preserving biologically meaningful marker-gene patterns [34]. Even when numerical improvements are moderate, the gains are statistically robust and translate into better separation of cell types and more stable downstream analyses. Overall, the results suggest that scDDI offers a balanced, interpretable, and reliable imputation strategy for diverse scRNA-seq applications.

## Materials and methods

### Formal details and background

The scDDI framework is structured around three key steps.

#### Calculation of dropout probabilities

Identifying dropout events accurately is essential for effective imputation in gene expression data. Zeros in the expression matrix can arise from two sources: technical dropouts, where a gene is expressed but not detected due to incomplete RNA capture, and biological zeros, where the gene is genuinely not expressed. Differentiating between these is challenging, especially for values close to zero.

scDDI employs a Poisson–negative binomial (PNB) mixture model to differentiate dropouts from true biological zeros. The Poisson component models dropout events, while the negative binomial (NB) component captures genuine gene expression. The PNB density function is defined as: 


(1) \begin{align*}& f_{\mathrm{PNB}}\left(y_{i} ; \lambda_{i}, \mu_{i}, \phi, \pi_{i}\right) = \pi_{i} f_{\mathrm{Pois}}\left(y_{i} ; \lambda_{i}\right) + (1 - \pi_{i}) f_{\mathrm{NB}}\left(y_{i} ; \mu_{i}, \phi \right),\end{align*}

where $\pi _{i}$ is the dropout probability for gene $g$ in cell $i$, $\lambda _{i}$ is the Poisson mean, and $\mu _{i}$ and $\phi $ denote the NB mean and dispersion parameters, respectively. To adjust for library size variability affecting dropout rates, scDDI models these parameters using generalized linear models with log-linear links [35].

The dropout probability $d_{i}$ for each observed count is computed as: 


(2) \begin{align*}& d_{i} = \frac{\hat{\pi}_{i} \hat{f}_{\mathrm{Pois}}\left(y_{i}; \hat{\lambda}_{i}\right)}{\hat{\pi}_{i} \hat{f}_{\mathrm{Pois}}\left(y_{i}; \hat{\lambda}_{i}\right) + (1 - \hat{\pi}_{i}) \hat{f}_{\mathrm{NB}}\left(y_{i}; \hat{\mu}_{i}, \hat{\phi}\right)},\end{align*}

where $\hat{\pi }_{i}$, $\hat{\lambda }_{i}$, $\hat{\mu }_{i}$, and $\hat{\phi }$ are parameters estimated via an EM algorithm [17]. Counts with $d_{i}> 0.5$ are classified as dropouts and subject to imputation.

#### Evaluation of cell-to-cell similarities

scDDI employs weighted cosine similarity (WCS) that integrates dropout probabilities into the conventional cosine similarity. Traditional cosine similarity measures the cosine of the angle between two nonzero vectors in an inner product space [36]. Incorporating dropout probabilities refines similarity estimation by accounting for missing values.

The WCS between cells $i$ and $j$ over $p$ genes is defined as: 


(3) \begin{align*}& s_{ij} = \frac{\sum_{g=1}^{p} w_{g} \, y_{gi} \, y_{gj}}{\sqrt{\sum_{g=1}^{p} w_{g} \, y_{gi}^{2}} \, \sqrt{\sum_{g=1}^{p} w_{g} \, y_{gj}^{2}}},\end{align*}

where the weight $w_{g} = 1$ if both $y_{gi}$ and $y_{gj}$ are either true expression or dropout values; otherwise, $w_{g}$ equals the dropout rate of gene $g$. This weighting scheme assigns lower weights to “questionable” gene pairs with low dropout rates, increasing similarity scores $s_{ij}$, while higher dropout rates yield larger weights, reducing $s_{ij}$. This reflects the assumption that dropout events in genes with low dropout frequency exert greater influence on cell-to-cell similarity.

#### Imputation using decision tree regression

scDDI employs decision tree regression [37] to impute missing gene expression values affected by dropout. The methodology comprises:

- **Input features:** Cells similar to the target cell are identified via WCS, with pairs exceeding a predefined threshold considered analogous. The regressor input includes WCS scores and observed gene expression values from these similar cells, based on the assumption that closely related cells exhibit correlated expression patterns.
- **Output features:** The model predicts imputed expression values for the gene in the target cell, reconstructing gene profiles by leveraging information from analogous cells to mitigate dropout-induced missingness.
- **Hyperparameter settings:** The model was implemented with DecisionTreeRegressor from scikit-learn [38]. Key hyperparameters, including maximum tree depth and minimum samples per leaf, were optimized via five-fold cross-validation to balance model complexity and prevent overfitting. In our experiments, the maximum tree depth was set to 15, and the minimum samples per leaf was set to 10, providing robust imputation performance across datasets
- **Model training and validation:** The decision tree regressor was trained on observed expressions of similar cells using a training-test split. Hyperparameters were optimized via five-fold cross-validation to prevent overfitting. Imputation accuracy was assessed on the test set using RMSE and Spearman correlation. The finalized model was then applied to impute missing values in the original expression matrix.

### Workflow

**Figure 1:**
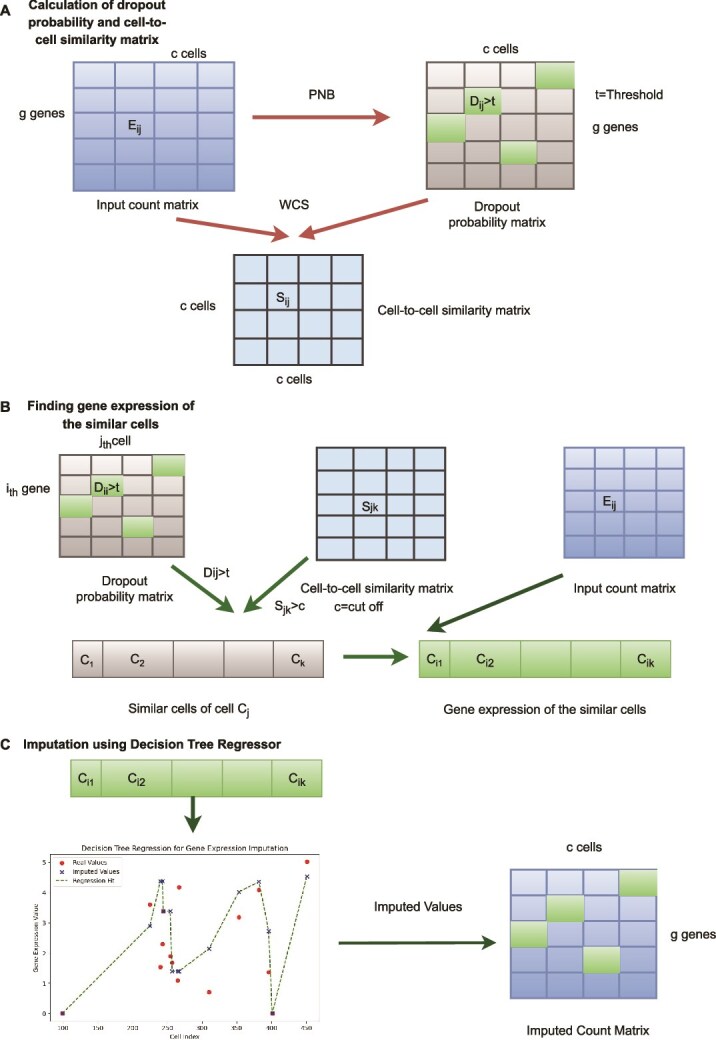
Overall workflow of scDDI: (A) Dropout probabilities are estimated using a PNB model from the preprocessed expression matrix. WCS is used to compute the cell-to-cell similarity matrix. (B) After detecting dropout at gene $g_{i}$ in cell $c_{j}$, similar cells of $c_{j}$ are identified and the expression values of gene $g_{i}$ for those cells are collected. (C) A decision tree regressor is used to impute the value of gene $g_{i}$ in cell $c_{j}$ based on the expression values of similar cells.

The workflow of the proposed scDDI method is illustrated in Fig. 1. Following subsections discussed the important steps:

#### Preprocessing of raw datasets

scRNA-seq datasets were obtained from publicly available repositories and represented as a matrix $ E_{g \times c} $, where $ g $ denotes the number of genes and $ c $ denotes the number of cells. Each element $ E_{ij} $ corresponds to the observed expression count of the $ i{\mathrm{th}} $ gene in the $ j{\mathrm{th}} $ cell. Quality control was performed by defining a cell as “high-quality” if it expressed >1000 genes, and a gene as “high-quality” if it exhibited a read count >5 in at least 5% of the cells in which it was expressed. The resulting filtered matrix $ E $, containing only high-quality genes and cells, was normalized using Linnorm [39], a normalization method based on linear modeling and normality transformation. The normalized matrix $ E^{\prime}_{g \times c} $ was subsequently log-transformed using a $ \log _{2} $ function with a pseudo-count of 1. To extract informative features for downstream analysis, the top 1000 highly variable genes (HVGs) were identified using Seurat’s HVG selection procedure [40].

#### Calculation of dropout probability matrix

We estimated the dropout probability for each cell–gene pair from the normalized gene expression matrix using the PNB mixture model. The resulting dropout probability matrix, $ D_{g \times c} $, comprises $ g $ genes and $ c $ cells, where each element $ D_{ij} $ represents the probability that the observed count of gene $ i $ in cell $ j $ is a dropout. A value of $ D_{ij}> 0.5 $ was considered indicative of a dropout event to be imputed. (See Fig. 1A for details.)

#### Calculation of cell-to-cell similarity matrix

Cell-to-cell similarity is computed via a WCS integrating normalized expression and dropout probabilities, yielding a similarity matrix $ S_{c \times c} $ where each element $ S_{ij} $ denotes similarity between cells $ c_{i} $ and $ c_{j} $. A dynamic cutoff is applied based on the sim.cut percentile of the similarity distribution (by default, the 75th percentile). Cells with similarity values above this threshold are considered sufficiently similar and are subsequently used for imputation. For each dropout event in gene $ g $ of a target cell, missing values are imputed using the expression profiles of the top $ k $ most similar cells that exceed the similarity cutoff. The parameter $ k $ is defined relative to the smallest expected subpopulation size: larger values of $ k $ are recommended when imputing within well-separated clusters, whereas smaller values are preferable when capturing fine-grained heterogeneity or rare subpopulations (Fig. 1A).

#### Finding gene expression of the similar cells

From the dropout probability matrix, cells requiring imputation within the gene expression matrix are identified. Specifically, for a given cell $ C_{j} $, if the dropout probability $ D_{ij} $ for gene $ i $ exceeds 0.5, the corresponding expression value $ E_{ij} $ is designated for imputation. Subsequently, similar cells to $ C_{j} $ are determined based on the cell-to-cell similarity matrix, and the expression values of gene $ i $ in these analogous cells, denoted as $ E_{i1}, E_{i2}, \ldots $, are retrieved to inform the imputation process. (See Fig. 1B for details.)

#### Imputation using decision tree regression model

Missing gene expression values in cells with identified dropout events are imputed using a decision tree regression approach. For each gene with a dropout event in the target cell, expression values from the most similar cells (e.g. $ E_{i1}, E_{i2} $, etc.) are leveraged based on the premise that closely related cells share correlated gene expression profiles. The observed expression values from these similar cells constitute the training dataset, while the missing values in the target cell form the test dataset. A decision tree regressor is trained on the observed data to model the relationship between gene expression values. Subsequently, this trained model is employed to predict the missing expression values in the target cell. The predicted values are then integrated into the original gene expression matrix as imputed values (Fig. 1C).

#### Algorithm

The procedural steps of scDDI are summarized in Algorithm 1.



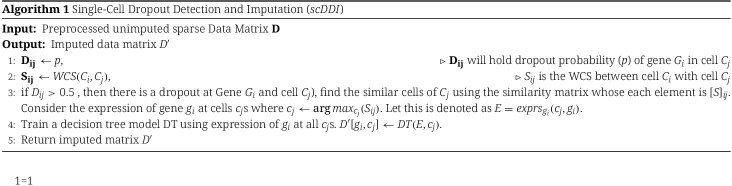



## Results and discussions

This section reports the empirical evaluation of scDDI across both simulated and real scRNA-seq datasets, with comparative benchmarking against state-of-the-art imputation approaches. Performance was assessed in terms of expression recovery (Pearson/Spearman correlations, RMSE, and MAE), clustering accuracy (ARI and NMI), and dropout detection (precision, recall, and F1-score). Statistical robustness was established through 95% confidence intervals and Wilcoxon signed-rank tests. In addition, visualization via UMAP embeddings and marker-gene analyses illustrates that scDDI preserves biologically meaningful structure and cellular heterogeneity.

### Clustering performance and expression recovery on simulated data

#### Clustering performance evaluation

We evaluated scDDI using seven synthetic scRNA-seq datasets generated with the splatSimulate function from the Splatter R package [41], designed to emulate realistic single-cell profiles with heterogeneous populations, variable gene expression, and dropout noise. Datasets were simulated with the “groups” method, predefined cell proportions, and specified proportions of differentially expressed (DE)genes [42]. Outlier genes were excluded by setting out.prob to zero. The configurations, spanning a range of cell-type complexities and expression dynamics, are summarized in Table 1.

**Table 1:** Summary of simulated datasets for clustering evaluation

**Dataset**	**Group proportions**	**DE proportions**	**No. of genes**	**No. of cells**
Dataset 1	(0.8, 0.1, 0.1)	(0.6, 0.2, 0.2)	5000	500
Dataset 2	(0.6, 0.2, 0.1, 0.1)	(0.5, 0.2, 0.2, 0.1)	8000	800
Dataset 3	(0.6, 0.2, 0.2)	(0.6, 0.3, 0.1)	6000	400
Dataset 4	(0.7, 0.2, 0.1)	(0.7, 0.2, 0.1)	10 000	600
Dataset 5	(0.8, 0.1, 0.1)	(0.5, 0.3, 0.2)	6000	2000
Dataset 6	(0.5, 0.2, 0.2, 0.1)	(0.4, 0.2, 0.2, 0.2)	10 000	5000
Dataset 7	(0.6, 0.2, 0.2)	(0.6, 0.2, 0.2)	8000	2000

We benchmarked scDDI against nine state-of-the-art imputation methods in addition to the raw data. Louvain clustering [43] was performed using Seurat [40], and clustering quality was assessed via ARI [31] and NMI [32]. As shown in Tables 2 and 3, scDDI consistently achieved higher ARI and NMI scores than the unimputed baseline and performed competitively with or better than leading benchmarks.

**Table 2:** ARI scores for raw and imputed data on simulated datasets

**Dataset**	**Raw**	**scDDI**	**scImpute**	**DrImpute**	**scDoc**	**scRMD**	**ALRA**	**MAGIC**	**SAVER**	**DeepImpute**	**scVI**
Dataset 1	0.160	**0.320**	0.190	0.210	0.130	0.200	0.150	0.100	0.160	0.305	0.14
Dataset 2	0.330	0.360	0.327	**0.500**	0.240	0.390	0.407	0.155	0.320	0.399	0.246
Dataset 3	0.526	0.540	0.450	0.570	0.370	0.449	0.320	0.180	0.430	**0.576**	0.23
Dataset 4	0.290	0.330	0.280	**0.365**	0.260	0.300	0.190	0.140	0.290	**0.365**	0.193
Dataset 5	0.088	0.110	0.090	0.180	0.110	0.100	**0.210**	0.070	0.130	0.170	0.07
Dataset 6	0.525	0.700	**0.736**	0.710	0.199	0.700	0.560	0.130	0.710	**0.736**	0.367
Dataset 7	0.288	**0.630**	0.498	0.617	0.270	0.480	0.460	0.120	0.500	0.360	0.193

**Table 3:** NMI scores for raw and imputed data on simulated datasets

**Dataset**	**Raw**	**scDDI**	**scImpute**	**DrImpute**	**scDoc**	**scRMD**	**ALRA**	**MAGIC**	**SAVER**	**DeepImpute**	**scVI**
Dataset 1	0.505	**0.630**	0.530	0.540	0.470	0.537	0.486	0.424	0.492	0.625	0.467
Dataset 2	0.690	0.700	0.688	**0.775**	0.610	0.730	0.735	0.540	0.685	0.720	0.63
Dataset 3	0.750	0.750	0.720	0.798	0.670	0.720	0.620	0.522	0.715	**0.799**	0.567
Dataset 4	0.614	0.632	0.594	0.660	0.584	0.620	0.520	0.465	0.614	**0.668**	0.53
Dataset 5	0.400	0.430	0.410	0.500	0.430	0.430	**0.510**	0.350	0.450	0.498	0.371
Dataset 6	0.750	0.877	**0.880**	**0.880**	0.580	0.877	0.780	0.520	**0.880**	0.670	0.697
Dataset 7	0.562	**0.820**	0.750	**0.820**	0.590	0.740	0.690	0.470	0.750	0.650	0.533

For ARI, scDDI raised Dataset 1 from 0.160 in the raw data to 0.320, achieved the highest score of 0.630 in Dataset 7, and performed strongly across the remaining datasets—ranking close behind DrImpute at 0.500 in Dataset 2 and DeepImpute at 0.576 in Dataset 3. In Dataset 6, DeepImpute and scImpute both reached 0.736, whereas scDDI achieved 0.700. Methods such as MAGIC and scDoc generally underperformed, especially under high dropout.

NMI results showed similar patterns. scDDI improved Dataset 1 from 0.505 to 0.630 and matched the highest score of 0.820 in Dataset 7. DeepImpute performed well in Dataset 3 with 0.799, while DrImpute reached 0.775 in Dataset 2. In contrast, scDoc, ALRA, and MAGIC exhibited consistently weaker NMI recovery. Across datasets, scDDI preserved biologically meaningful structures and sharpened separation between closely related subpopulations, supporting rare-cell detection [44] under dropout noise.

**Figure 2:**
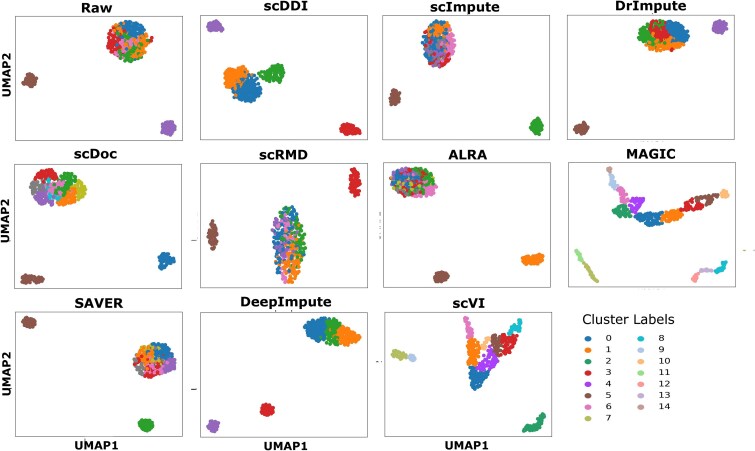
Comparison of clustering results on the imputed Simulated Dataset 1 across eleven methods: the raw data, scDDI, and nine other state-of-the-art approaches.

Figure 2 presents UMAP [33] visualizations of Dataset 1 for the raw data and after imputation with scDDI and comparison methods, illustrating that scDDI yields clearer and more coherent clustering boundaries. An analogous UMAP visualization for Dataset 7, demonstrating the effect of scDDI and competing methods on a larger simulated dataset, is presented in Supplementary Fig. S1. Overall, scDDI substantially enhances clustering accuracy and improves the delineation of cell subpopulations [45] in simulated datasets, particularly under moderate to high dropout levels. By maintaining a balance between dropout correction and biological signal preservation, scDDI not only rivals or surpasses DeepImpute and DrImpute but also consistently outperforms ALRA, MAGIC, scDoc, and scVI. These results underscore scDDI’s robustness in recovering fine-grained cellular heterogeneity from noisy single-cell data.

#### Expression recovery

To evaluate the robustness and accuracy of our proposed imputation method under varying dropout conditions, we simulated six scRNA-seq datasets with the widely used R package Splatter [41], with controlled dropout rates of 30%, 40%, 50%, 60%, 70%, and 80%. The simulation included group structures and DE [42] genes to mimic realistic biological scenarios. Dropout was manually applied to the raw count matrix to precisely control sparsity, while the original, unperturbed count matrix was retained as the ground truth.

**Figure 3:**
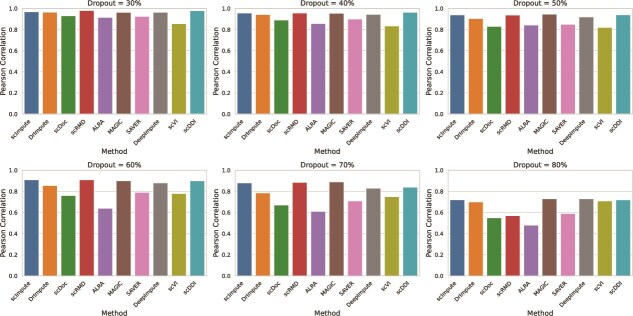
Pearson correlation between the real and imputed values of the simulated data under different dropout rates, where higher correlation coefficients indicate better imputation performance.

**Figure 4:**
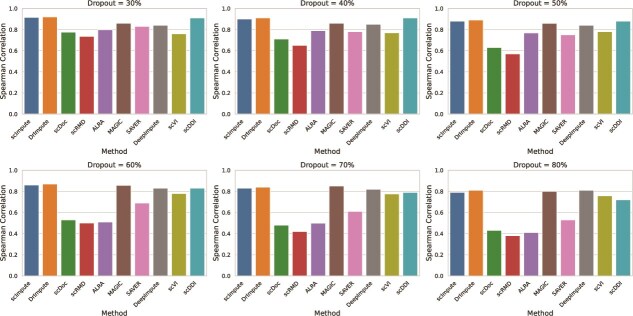
Spearman rank correlation between the real and imputed values of the simulated data under different dropout rates, where higher correlation coefficients indicate better imputation performance.

Imputation accuracy was quantitatively evaluated using the Pearson correlation coefficient, measuring the degree of linear agreement between imputed and ground truth expression values, and the Spearman rank correlation coefficient, evaluating the monotonic consistency of gene expression recovery irrespective of linearity. To assess the comparative performance of the proposed method, Pearson and Spearman correlation coefficients were computed for scDDI and nine state-of-the-art imputation methods—scImpute [12], DrImpute [7], scDoc [15], scRMD [21], ALRA [19], MAGIC [9], SAVER [13], DeepImpute [26], and scVI [27] across all simulated dropout levels. The comparative results are summarized as Pearson and Spearman bar plots in Figs 3 and 4, respectively. The corresponding numerical values for Pearson and Spearman correlations are provided in Supplementary Tables S1 and S2, respectively.

From these figures, it is evident that both Pearson and Spearman correlation coefficients declined across all methods as the dropout rate increased. Nevertheless, the proposed method, scDDI, consistently achieved higher correlations than most competitors. For example, at a dropout rate of 40%, scDDI reached a Pearson correlation of 0.964 and a Spearman correlation of 0.91. Even under severe dropout at 80%, scDDI maintained a Pearson correlation of 0.72 and a Spearman correlation of 0.72, surpassing the majority of alternative approaches.

Overall, two bar plots clearly demonstrate the robustness and reliability of scDDI, particularly under high levels of sparsity. Its consistently strong performance across both correlation measures suggests substantial potential for application in real-world scRNA-seq analyses, where dropout remains a pervasive challenge.

### Clustering performance and expression recovery on real-life scRNA-seq datasets

#### Overview of datasets

We have used seven public benchmark scRNA-seq datasets: Yan [46], Ting [47], Goolam [48], Pollen [49], Darmanis [50], Melanoma [51], and PBMC [52]. Table 4 shows a summary of the used datasets. A detailed description of these datasets is provided in Supplementary Section S2.2. We retain these data to know the efficacy of our method in both small and large scRNA-seq data.

**Table 4:** A brief summary of the real scRNA sequence dataset

**Dataset name**	**Description**	**Features (genes)**	**Instances (cells)**	**Class**
Yan [46]	Human embryo cell	20 214	90	7
Ting [47]	Pancreatic tumor cell	29 018	187	7
Goolam [48]	Mouse embryo cell	41 480	124	5
Pollen [49]	Human tissues	23 794	299	11
Darmanis [50]	Human brain cell	22 088	466	9
Melanoma [51]	Human tumor cell	19 783	68 579	14
PBMC [52]	Blood mononuclear cell	33 694	4340	8

#### Clustering performance evaluation

**Figure 5:**
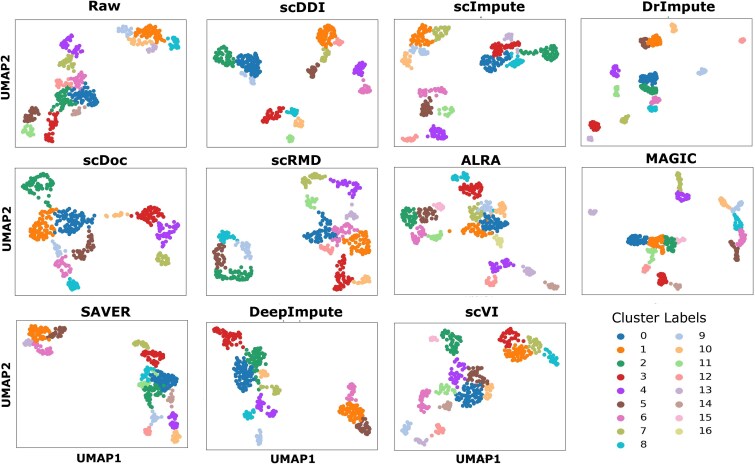
Comparison of clustering results on the imputed Darmanis dataset across eleven methods: the raw data, scDDI, and nine other state-of-the-art approaches.

We assessed the impact of scDDI on downstream clustering performance across seven benchmark scRNA-seq datasets spanning diverse tissues and sequencing platforms. The evaluation included scDDI, nine state-of-the-art imputation methods, and the unimputed datasets. Clustering quality was quantified using the ARI and NMI, as summarized in Tables 5 and 6. Figure 5 presents UMAP visualizations of Darmanis dataset for the raw data and after imputation with scDDI and state-of-art methods. In addition, the corresponding UMAP visualization for Goolam dataset is provided in Supplementary Fig. S2.

**Table 5:** ARI scores for raw and imputed data on real datasets

**Dataset**	**Raw**	**scDDI**	**scImpute**	**DrImpute**	**scDoc**	**scRMD**	**ALRA**	**MAGIC**	**SAVER**	**DeepImpute**	**scVI**
Darmanis	0.26	0.344	0.265	0.28	0.322	0.266	0.23	0.23	0.29	**0.377**	0.348
Goolam	0.398	**0.545**	0.535	0.44	0.53	0.39	0.39	0.459	0.45	0.447	0.448
Pollen	0.34	0.699	0.654	0.64	0.605	0.699	**0.831**	0.817	0.698	0.639	0.642
Yan	0.84	0.842	**0.857**	0.80	0.85	0.84	0.69	0.69	0.845	0.79	0.622
Ting	0.43	0.51	0.46	0.43	0.43	0.514	0.385	0.32	0.48	**0.52**	0.304
Melanoma	0.31	0.36	0.24	**0.40**	0.31	0.23	0.348	0.21	0.35	0.16	0.306
PBMC	0.43	**0.54**	0.48	0.407	0.43	0.47	0.50	0.19	0.324	0.42	0.436

**Table 6:** NMI scores for raw and imputed data on real datasets

**Dataset**	**Raw**	**scDDI**	**scImpute**	**DrImpute**	**scDoc**	**scRMD**	**ALRA**	**MAGIC**	**SAVER**	**DeepImpute**	**scVI**
Darmanis	0.49	0.51	0.48	0.487	0.525	0.49	0.495	0.486	0.508	**0.545**	0.528
Goolam	0.68	**0.79**	0.77	0.729	0.77	0.68	0.67	0.733	0.745	0.744	0.731
Pollen	0.58	0.888	0.845	0.85	0.818	0.881	0.907	**0.908**	0.88	0.85	0.837
Yan	0.86	0.873	**0.888**	0.83	0.88	0.87	0.847	0.81	0.86	0.83	0.787
Ting	0.646	**0.76**	0.65	0.685	0.69	0.718	0.62	0.53	0.69	0.73	0.533
Melanoma	0.585	0.611	0.52	**0.63**	0.51	0.52	0.598	0.55	0.605	0.294	0.557
PBMC	0.674	**0.702**	0.67	0.638	0.63	0.68	0.66	0.51	0.605	0.65	0.656

Across most datasets, scDDI substantially improved clustering accuracy compared with the unimputed baseline and frequently outperformed or matched the strongest alternatives. In Goolam, it achieved the highest ARI of 0.545 and NMI of 0.79, while in Ting it reached the highest NMI of 0.76 with a competitive ARI of 0.51, surpassing ALRA, MAGIC, and scImpute.

In Darmanis, DeepImpute obtained the top ARI of 0.377 and NMI of 0.545, with scDDI close behind at 0.344 and 0.51, preserving biological structure. In Pollen, ALRA reached the best ARI of 0.831 and MAGIC the best NMI of 0.908, yet scDDI maintained strong performance with an ARI of 0.699 and NMI of 0.888 across this heterogeneous dataset. In Yan, scDDI delivered an ARI of 0.842 and NMI of 0.873, ranking just below the leaders but still providing meaningful clustering resolution.

Importantly, in PBMC scDDI achieved the highest scores with an ARI of 0.54 and NMI of 0.702, clearly enhancing the resolution of closely related subpopulations. Conventional methods such as scImpute, DrImpute, and scRMD often produced inconsistent results, while deep learning-based approaches like DeepImpute excelled in some datasets but lacked consistent superiority.

Overall, scDDI consistently improved clustering accuracy and strengthened the identification of biologically meaningful cell subpopulations [14] in real datasets, while preserving marker gene expression patterns essential for interpretation. These combined strengths establish scDDI as a robust and generalizable imputation framework for diverse scRNA-seq applications.

#### Expression recovery

**Figure 6:**
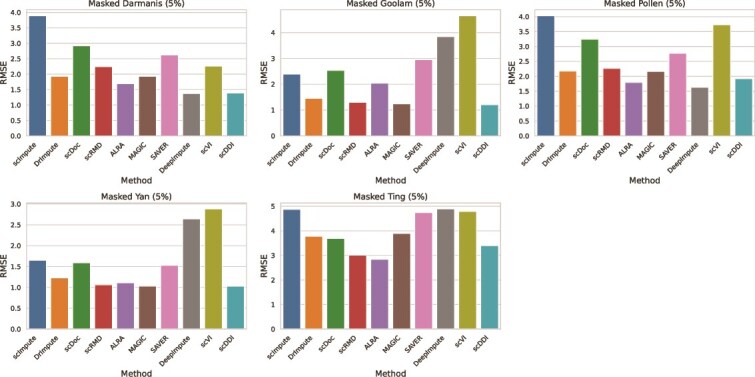
Imputation accuracy on six real datasets measured by RMSE between the imputed values and real values, lower RMSE indicate better performance.

**Figure 7:**
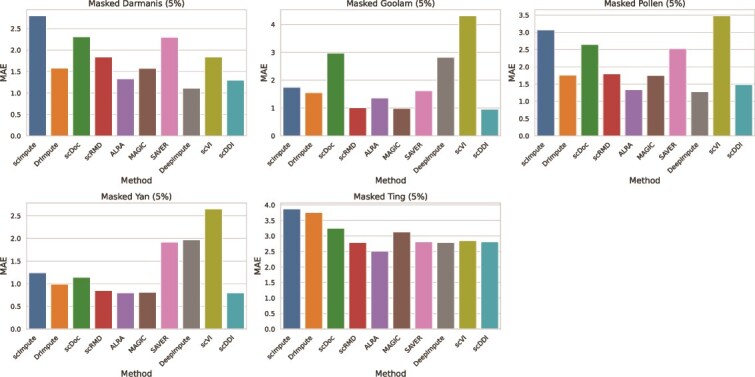
Imputation accuracy on six real datasets measured by MAE between the imputed values and real values, lower MAE indicate better performance.

To evaluate the effectiveness of the imputation methods in recovering masked gene expression values, we calculated the RMSE and MAE across five real scRNA-seq datasets—Darmanis, Goolam, Pollen, Yan, and Ting—each with 5% of nonzero entries artificially masked. The comparative results are summarized as RMSE and MAE bar plots in Figs 6 and 7, which clearly illustrate method-specific differences in recovery accuracy. The corresponding numerical values for RMSE and MAE are provided in Supplementary Tables S3 and S4, respectively.

Across all datasets, scDDI consistently achieved among the lowest RMSE and MAE values, reflecting superior accuracy in recovering masked expression. On the Masked Goolam dataset, scDDI recorded an RMSE of 1.21 and an MAE of 0.96, outperforming all other methods. In the Masked Yan dataset, it attained the best MAE of 0.80 together with one of the lowest RMSE values of 1.03, demonstrating robustness in compact, low-expression settings. In the Masked Darmanis dataset, scDDI achieved an RMSE of 1.39 and an MAE of 1.30, again improving upon most competitors.

DeepImpute and DrImpute also showed competitive performance in several datasets, particularly Goolam and Yan, yet were frequently surpassed by scDDI. In contrast, methods such as MAGIC, scImpute, and scDoc generally produced higher RMSE and MAE values, suggesting less effective recovery under sparse conditions.

Overall, the bar plots clearly demonstrate that scDDI not only improves clustering and dropout detection, but also excels at expression recovery, consistently delivering accurate reconstruction of masked values across diverse real datasets.

### Ablation study

To investigate the contribution of each component in scDDI, we conducted a comprehensive ablation study across all real datasets used in our evaluation. The proposed scDDI framework integrates three major modules: (i) dropout probability estimation using the PNB model, (ii) WCS for computing inter-cell similarity, and (iii) Decision Tree Regression for final imputation of dropout-prone gene expressions.

To evaluate the contribution of each component of scDDI, we conducted an ablation study in which individual modules were systematically removed or substituted, resulting in eight model variants. These included: (i) PNB–WCS–Regressor (the complete scDDI model), (ii) PNB–WCS–Mean (Decision Tree Regressor replaced by mean imputation), (iii) PNB–Std–Regressor (WCS replaced by standard cosine similarity), (iv) PNB–Std–Mean, (v) Log–WCS–Regressor (PNB replaced by a logistic dropout approximation), (vi) Log–WCS–Mean, (vii) Log–Std–Regressor, and (viii) Log–Std–Mean. Each variant was systematically evaluated using ARI and NMI on six real scRNA-seq datasets—Darmanis, Goolam, Pollen, Yan, Ting, and PBMC—to rigorously quantify the individual contributions of the dropout model, similarity measure, and regression module.

The clustering results, measured by the ARI, are summarized in Table 7. The complete model (PNB–WCS–Tree) consistently achieves the highest ARI in all datasets, confirming that each component contributes significantly to the overall performance of scDDI. Removing any single module led to a decrease in clustering accuracy, with the largest performance drop observed when PNB-based dropout modeling or decision tree regression was omitted. This indicates that accurate dropout probability estimation and nonlinear imputation are critical for capturing cell-specific transcriptional variability. For completeness, the NMI results corresponding to the same ablation settings are provided in Supplementary Table S5, which exhibits trends consistent with the ARI analysis.

**Table 7:** Results of ablation study of **scDDI** on six real datasets measured by ARI

**PNB**	**WCS**	**Regressor**	**Darmanis**	**Goolam**	**Pollen**	**Yan**	**Ting**	**PBMC**
✓	✓	✓	**0.34**	**0.545**	**0.699**	**0.84**	**0.51**	**0.54**
✓	✓	$\times $	0.33	0.38	0.66	0.81	0.43	0.51
✓	$\times $	✓	0.30	0.53	0.66	0.80	0.43	0.52
✓	$\times $	$\times $	0.28	0.38	0.63	0.80	0.32	0.51
$\times $	✓	✓	0.30	0.54	0.30	0.83	0.44	0.50
$\times $	✓	$\times $	0.28	0.39	0.68	0.79	0.46	0.48
$\times $	$\times $	✓	0.27	0.53	0.66	0.80	0.43	0.49
$\times $	$\times $	$\times $	0.27	0.40	0.63	0.79	0.36	0.46

*Note.* ✓ indicates that the component is included; $\times $ indicates that it is removed or replaced. The full model (✓ ✓ ✓; i.e. **PNB–WCS–Regressor**) consistently achieves the highest clustering performance across all datasets.

Taken together, the ablation analysis indicates that the integrated contribution of probabilistic dropout modeling, weighted similarity computation, and regression-driven imputation constitutes the core mechanism underlying the robustness and high performance of scDDI.

### Statistical validation of clustering performance

We evaluated post-imputation clustering using the ARI and NMI, two standard measures of concordance between inferred clusters and known cell-type labels. For each method, we reported mean values with 95% confidence intervals [53] to capture robustness across datasets. To test significance, we employed the Wilcoxon signed-rank test [54], a nonparametric paired procedure, comparing all methods against scDDI as the reference. This framework ensured that improvements attributed to scDDI were not only consistent but also statistically meaningful.

As shown in Table 8, scDDI achieved the highest mean ARI of 0.549 $\pm $ 0.164, significantly outperforming scImpute, DrImpute, SAVER, scDoc, and scVI. Similarly, in Table 9, scDDI obtained the highest mean NMI of 0.733 $\pm $ 0.127, with significant gains over scImpute, DrImpute, scRMD, MAGIC, SAVER, DeepImpute, and scVI.

**Table 8:** ARI results (Mean $\pm $ 95% CI) and Wilcoxon signed-rank test $P$-values vs. scDDI

**Method**	**ARI Mean $\pm $ CI**	** $P$ -value vs. scDDI**
scDDI	0.549 $\pm $ 0.164	–
scImpute	0.499 $\pm $ 0.199	0.0469
DrImpute	0.485 $\pm $ 0.162	0.0312
scDoc	0.497 $\pm $ 0.174	0.0312
scRMD	0.487 $\pm $ 0.205	0.0938
ALRA	0.482 $\pm $ 0.194	0.1562
MAGIC	0.417 $\pm $ 0.231	0.0781
SAVER	0.491 $\pm $ 0.192	0.0469
DeepImpute	0.479 $\pm $ 0.185	0.0781
scVI	0.444 $\pm $ 0.130	0.0312

**Table 9:** NMI results (Mean $\pm $ 95% CI) and Wilcoxon signed-rank test $P$-values vs. scDDI

**Method**	**NMI Mean $\pm $ CI**	** $P$ -value vs. scDDI**
scDDI	0.733 $\pm $ 0.127	–
scImpute	0.689 $\pm $ 0.144	0.0312
DrImpute	0.693 $\pm $ 0.116	0.0312
scDoc	0.689 $\pm $ 0.132	0.0781
scRMD	0.691 $\pm $ 0.141	0.0156
ALRA	0.685 $\pm $ 0.133	0.0781
MAGIC	0.647 $\pm $ 0.156	0.0312
SAVER	0.699 $\pm $ 0.128	0.0156
DeepImpute	0.663 $\pm $ 0.179	0.0469
scVI	0.661 $\pm $ 0.117	0.0312

These results confirm that scDDI consistently delivers more accurate and coherent clustering than competing imputation methods. Results for simulated datasets are provided in the Supplementary Tables S6 and S7.

### Sensitivity analysis for selection of dropout threshold

We evaluated the ability of scDDI to accurately detect dropout events using generated simulated scRNA-seq datasets, where the true dropout positions are known. Dropout prediction in scDDI is based on the estimated dropout probability matrix $ d_{ij} $, where each entry quantifies the likelihood that the observed zero at gene $ i $ in cell $ j $ is a dropout. A binary decision was made by applying a threshold $\tau $, such that values with $ d_{ij}> \tau $ were classified as dropouts. Thresholds were systematically varied from 0.3 to 0.8 in increments of 0.1 to examine the sensitivity of detection performance. For each threshold, predicted dropout events were compared against the ground truth, and we computed three complementary evaluation metrics: precision, recall, and the F1 score. These metrics collectively provide a balanced view of model performance, capturing both accuracy and sensitivity.

**Figure 8:**
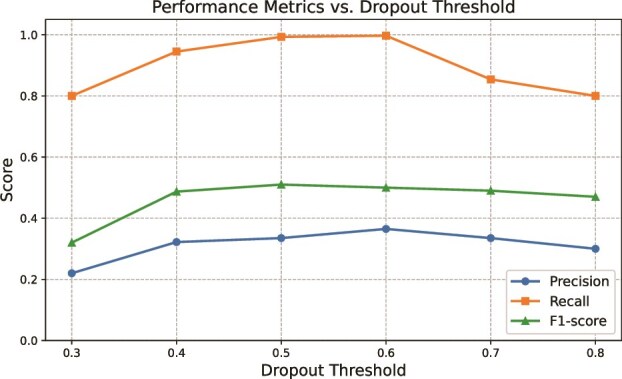
Performance metrics (precision, recall, and F1-score) for dropout identification across varying dropout probability thresholds, where higher values indicate better agreement between predicted and true dropout events.

The results demonstrate that scDDI consistently achieves high precision, recall, and F1 scores across the full range of thresholds, indicating robustness in dropout detection. The best trade-off between precision and recall was observed around $\tau = 0.5$, where the F1 score reached its maximum. This suggests that a moderate threshold strikes an effective balance between avoiding false positives and recovering true dropouts. Figure 8 summarizes the performance trends, highlighting scDDI’s stable accuracy across varying decision thresholds.

### Validation of imputed data in downstream analysis

**Figure 9:**
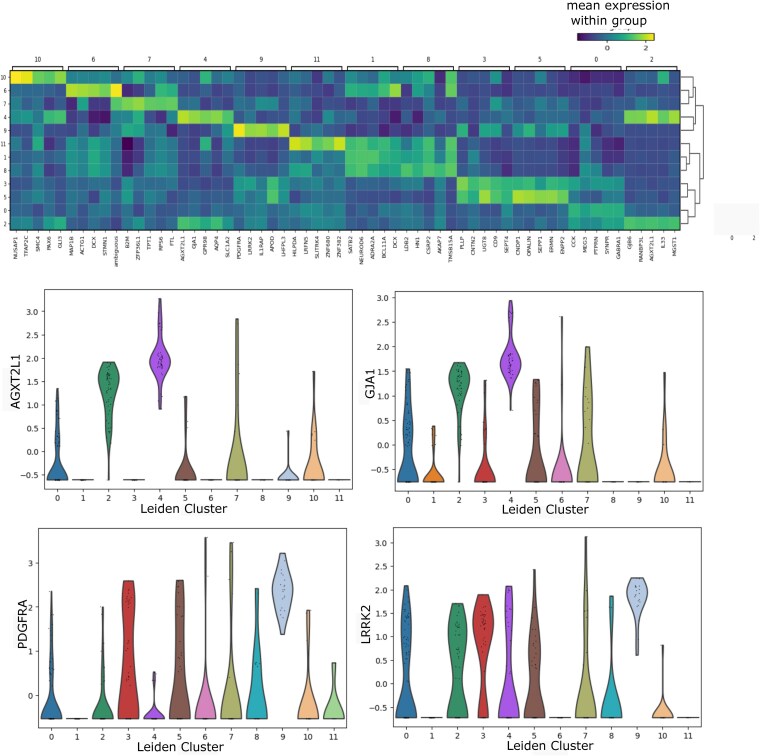
Marker gene analysis for the imputed Darmanis dataset showing a heatmap of average expression values of the top five differentially expressed genes and violin plots of their expression profiles across clusters.

To assess the influence of scDDI-imputed data on downstream analyses, we conducted clustering and marker gene identification [34] on the imputed Darmanis dataset. Differential expression analysis was performed using the Wilcoxon rank-sum test [54], and the top five marker genes for each cluster were identified. We visualized these top DE genes using a matrix plot, where the mean expression of each gene within each cluster is represented as a heatmap. The resulting marker gene patterns are shown in Fig. 9. For example, AGXT2L1 and GJA1 exhibited pronounced overexpression in cluster 4, whereas PDGFRA and LRRK2 showed elevated expression in cluster 9. These results demonstrate that scDDI effectively preserves biologically meaningful signals after imputation. Additionally, violin plots of selected marker genes across Leiden clusters for imputed Pollen dataset is provided in the Supplementary Fig. S4.

### Computational considerations

scDDI consists of a preprocessing step for dropout probability estimation and cell-to-cell similarity calculation, followed by decision tree regressor-based imputation. While the preprocessing step is relatively more demanding, it is performed only once and its outputs can be reused across downstream analyses. The imputation step itself is lightweight, making the framework practical for both medium and large-scale datasets when run on standard desktop systems or high-performance servers.

All experiments were run both on a high-performance server for large datasets and on a system with Ubuntu 22.04.5 LTS, 12th Gen Intel$\circledR $ Core™ i5-1240P (12 cores, 16 threads), and 16 GB RAM. For larger real datasets such as Melanoma, PBMC, and larger simulated datasets (Dataset 5, Dataset 6, and Dataset 7), which we processed using a dedicated high-performance server to accommodate the computational demands.

## Conclusions

Dropout events represent a substantial challenge in the analysis of scRNA-seq data. Many existing computational methods inadequately address these missing values, potentially introducing bias and compromising downstream analyses. To address this, we developed scDDI, an imputation framework that integrates weighted cell–cell similarity with decision tree regression to accurately detect and impute dropouts, enabling faithful reconstruction of the cell–gene expression matrix. scDDI uses the WCS, incorporating gene-specific weights derived from dropout probabilities to refine similarity estimation. Imputation is then performed using decision tree regression trained on highly similar cells, facilitating the recovery of biologically meaningful expression values.

We performed comprehensive benchmarking on seven real and seven simulated scRNA-seq datasets, systematically comparing scDDI with eight state-of-the-art imputation methods. Our evaluation encompassed diverse performance metrics including expression recovery (Pearson and Spearman correlations, RMSE, andMAE), clustering quality (ARI and NMI), and dropout identification accuracy (precision, recall, and F1-score) across varying dropout thresholds. Statistical rigor was ensured through 95% confidence intervals and Wilcoxon signed-rank teststhat collectively demonstrated the consistent superiority or competitive performance of scDDI across heterogeneous datasets and evaluation criteria.

These findings highlight scDDI’s capacity to reconstruct biologically relevant gene expression patterns while preserving cellular heterogeneity, thereby enhancing the robustness and interpretability of downstream analyses such as clustering and differential expression.

Nonetheless, some limitations persist. The performance of scDDI is dependent on parameter settings, including the similarity cutoff and the number of neighboring cells used for imputation, which may require optimization tailored to specific datasets and biological contexts. Future work will focus on adaptive parameter tuning and extending the framework to accommodate emerging single-cell multi-omics data modalities. We also plan to incorporate additional cell-level features (e.g. cell-type annotations, spatial coordinates, or epigenetic profiles) into the similarity calculation when available, to further enhance robustness and biological relevance. In addition, to demonstrate broader applicability beyond clustering, we will systematically evaluate scDDI in other key downstream analyses—specifically trajectory inference and gene regulatory network reconstruction in future work.

In summary, scDDI represents a robust and statistically validated framework for scRNA-seq imputation, offering an effective balance between accuracy, interpretability, and generalizability. By consistently reconstructing biologically meaningful gene expression patterns, scDDI establishes a reliable benchmark for single-cell data recovery and downstream analysis.

**Key Points**

- Single-cell RNA sequencing (scRNA-seq) provides a powerful means to analyze gene expression patterns at the single-cell level, yet dropout events lead to substantial zero counts in expression matrices, prompting the need for robust imputation methods.
- Introducing single-cell dropout detection and imputation (scDDI): A novel approach leveraging Poisson-Negative Binomial mixture models, cell-to-cell similarity calculations, and decision tree regression for accurate detection and imputation of dropout events in scRNA-seq data.
- Evaluation on simulated and real datasets demonstrates the superiority of scDDI over established imputation techniques, significantly enhancing performance in cell clustering, differential expression analysis, and downstream tasks.
- scDDI offers a comprehensive solution by effectively addressing technical and biological dropout events, paving the way for improved scRNA-seq analysis.

## Supplementary Material

scDDI_supplementary_bbag072

## Data Availability

All the data and codes (scDDI R package) are available in the github page https://github.com/AFMShamsuzzaman/scDDI. A demo running of scDDI with example input and output data is also provided in the github page https://github.com/AFMShamsuzzaman/Introduction-to-scDDI/.
