# Supplementary material for: Imputing missing values in single-cell RNA-sequencing data: a statistical and machine learning-based approach
Source: Brief Bioinform. 2026 Feb 16;27(1):bbag072. doi: 10.1093/bib/bbag072 (PMC12908672; doi:10.1093/bib/bbag072)
Supplement: scDDI_supplementary_bbag072 [file scddi_supplementary_bbag072.pdf]

Supplementary information for the article: “Imputing Missing Values in Single-Cell RNA-seq Data: A statistical and machine learning based approach”

AFM Shamsuzzaman[a], Sumanta Ray[b*], Anirban Mukhopadhyay[c*]
[a] Department of Computer Science, Raja Rammohun Roy Mahavidyalaya,affiliated to University of Burdwan , Radhanagar, Hooghly, West Bengal, India
[b] Department of Data Science and Data Protection, The West Bengal National University of Juridical Sciences, Kolkata, India
[c]Department of Computer Science and Engineering, University of Kalyani, Kalyani, Nadia, West Bengal, India

S1 Supplementary methods

S1.1 Data Preprocessing

Publicly available single-cell RNA sequencing datasets, typically in raw `.rds` or `.mtx` formats, were filtered to retain high-quality cells and genes. Cells expressing over 1000 genes were considered high-quality, while genes with a minimum count greater than 5 in at least 5% of cells were retained. The filtered expression matrix was normalized using the Linnorm[1] method, which applies a linear model and normality-based transformation, followed by a $\log_2$ transformation with a pseudocount of one. Feature selection employed Seurat’s highly variable gene (HVG) approach [2] to identify the top 1000 genes for downstream analysis.

S1.2 Dropout Probability Estimation

scDDI models observed zeros in scRNA-seq data using a Poisson–Negative Binomial (PNB) mixture model, which distinguishes technical dropouts from true biological absences. The Poisson component captures dropout events, while the Negative Binomial component models genuine gene expression:

$$f_{PNB}(y_i; \lambda_i, \mu_i, \phi, \pi_i) = \pi_i f_{Pois}(y_i; \lambda_i) + (1 - \pi_i) f_{NB}(y_i; \mu_i, \phi), \tag{1}$$

where π_i is the dropout probability for gene g in cell i, and λ_i, μ_i, and ϕ are Poisson mean, NB mean, and NB dispersion parameters, respectively. Library size effects are accounted for using generalized linear models (GLMs) [3]:

$$\log(\lambda_i) = \gamma + \log N_i, \quad \log(\mu_i) = \alpha + \log N_i, \quad \log(\phi) = \delta, \tag{2}$$

with N_i as the total counts in cell i. Dropout probability is computed as:

$$d_i = \frac{\hat{\pi}_i f_{Pois}(y_i; \hat{\lambda}_i)}{\hat{\pi}_i f_{Pois}(y_i; \hat{\lambda}_i) + (1 - \hat{\pi}_i) f_{NB}(y_i; \hat{\mu}_i, \hat{\phi})}, \tag{3}$$

where parameters are estimated via the EM algorithm [4]. Counts with $d_i > 0.5$ are classified as dropouts for imputation.

S1.3 Cell-to-cell similarity calculation

scDDI computes cell-to-cell similarity using Weighted Cosine Similarity (WCS), which integrates drop-out probability into the standard cosine similarity framework. For two m-dimensional non-zero vectors A and B, cosine similarity is defined as:

$$\cos(\theta) = \frac{\sum_{l=1}^{m} A_l B_l}{\sqrt{\sum_{l=1}^{m} A_l^2}\sqrt{\sum_{l=1}^{m} B_l^2}}. \tag{4}$$

WCS between cells i and j over p genes is given by:

$$s_{ij} = \frac{\sum_{g=1}^{p} w_g\, y_{gi}\, y_{gj}}{\sqrt{\sum_{g=1}^{p} w_g\, y_{gi}^2}\sqrt{\sum_{g=1}^{p} w_g\, y_{gj}^2}}, \tag{5}$$

where $w_g = 1$ if y_{gi} and y_{gj} are both non-drop-out or both drop-out values; otherwise w_g equals the gene's drop-out rate. This weighting reduces the influence of uncertain measurements.

S1.4 Imputation Using Decision Tree Regression

Missing gene expression values are imputed using a decision tree regression model [5]. First, cells similar to the target cell (with detected dropout) are identified via the Weighted Cosine Similarity (WCS) metric. Cells with WCS exceeding a predefined threshold are considered similar, and their gene expression profiles serve as input features for the regressor [6]. The similar-cell data are partitioned into training and test sets. The training set, comprising known expression values, is used to fit the decision tree model, which then predicts the missing values for the test set. These predictions are integrated into the target cell's expression profile, improving the accuracy of data reconstruction.

S2 Supplmentary Data

S2.1 Generation of Simulated data

We generated two categories of simulated datasets using the Splatter R package to evaluate expression recovery and clustering performance of the proposed scDDI method.

S2.1.1 Simulated Datasets for Expression Recovery Evaluation

To evaluate expression recovery accuracy, we computed Pearson correlation and Spearman rank correlation between recovered and ground-truth expression values. Six datasets with varying dropout rates were generated.

We first simulated datasets without dropout, but with predefined group structures and differentially expressed (DE) genes. Then, manual dropout was applied at target rates of 30%, 40%, 50%, 60%, 70%, and 80%. For each dataset, we also recorded the observed dropout rate after simulation to ensure reproducibility.More specifically, the datasets can be generated with the following R codes:

```
library(splatter)
library(SingleCellExperiment)

# Step 1: Simulate base dataset without dropout
params <- newSplatParams()
params <- setParams(params,
  group.prob = c(0.6, 0.3, 0.1), # Cell group proportions
  de.prob = c(0.3, 0.5, 0.8),    # DE probability per group
  de.facLoc = 0.4,              # Mean log fold change
  de.facScale = 0.8,          # Variability in log fold change
  batchCells = 200,             # Number of cells
  nGenes = 5000,                # Number of genes
  dropout.type = "none"         # Disable built-in dropout
)
sim <- splatSimulate(params, verbose = FALSE, seed = 123)
true_counts <- assay(sim, "counts")

# Step 2: Function to apply manual dropout
add_dropout <- function(count_matrix, rate, seed = 1) {
  set.seed(seed)
  mask <- matrix(runif(length(count_matrix)) < rate,
                 nrow = nrow(count_matrix),
                 ncol = ncol(count_matrix))
  dropped <- count_matrix
  dropped[mask & count_matrix > 0] <- 0
  return(dropped)
}

# Step 3: Apply dropout at target rates
dropout_rates <- c(0.3, 0.4, 0.5, 0.6, 0.7, 0.8)
names(dropout_rates) <- paste0("dropout", dropout_rates * 100)
```

S2.1.2 **Simulated Datasets for Clustering Performance Evaluation**

For clustering performance assessment, we generated seven simulated datasets with different numbers of cells, genes, group proportions, and DE proportions. The clustering performance was evaluated using Adjusted Rand Index (ARI) and Normalized Mutual Information (NMI). These datasets can be generated with the following R codes:

```
library(splatter)
library(SingleCellExperiment)
dataset1 <- splatSimulate(
    group.prob = c(0.8, 0.1, 0.1), # Cell group proportions
    method = 'groups',             # Simulation method
    batchCells = 500,            # Total number of cells
    de.prob = c(0.6, 0.2, 0.2), # Proportion of DE genes for each group
```

```
    de.facLoc = 0.4,          # Mean log fold change
    de.facScale = 0.8,       # Variability in log fold change
    nGenes = 5000,           # Total number of genes
    out.prob = 0,            # Proportion of outlier genes
    verbose = FALSE          # Suppress detailed output messages
)
#Extract observed and true (pre-dropout) count matrices
observed_counts <- counts(dataset1)
true_counts <- assays(dataset1)$TrueCounts
```

S2.2 Real scRNA-seq Datasets

Seven single-cell RNA-seq real datasets are used for the evaluation of the proposed method: Yan, Ting, Goolam, Pollen, Darmanis, Melanoma,and PBMC.

- Yan [7]: This is a human preimplantation embryo and embryonic stem cell dataset. There are 7 cell types, including labelled 4-cell,8-cell, zygote, Late blastocyst and 16-cell.[GEO under accession no. GSE36552].
- Ting [8] : It is circulating Tumor Cells (CTCs) are shed from primary tumors into the bloodstream, mediating the hematogenous spread of cancer to distant organs. It is downloaded from GEO under accession no. GSE51372.
- Goolam [9]: The dataset consists of Single-cell RNA-seq of blastomeres from 2- to 32-cell stage mouse embryos. It contains five cell types.
- Pollen [10] : This single cell RNA seq pair-end 100 reads from single cell cDNA libraries were quality trimmed. It contains eleven cell types. [GEO accession no GEO1832359].
- Darmanis [11]: This single cell RNA sequencing dataset is taken on 466 cells to capture the cellular complexity of the adult and fetal human brain at a whole transcriptome level. It contains nine cell types.[GEO accession no GSE67835]
- Melanoma [12]:The dataset describes the diversity of expression states within melanoma tumors, it is obtained freshly resected samples, disaggregated the samples, sorted into single cells, and profiled them by single-cell RNA-seq. It is downloaded from GEO under accession no. GSE72056.
- PBMC4K [13]: It is Peripheral blood mononuclear cells (PBMCs) from a healthy donor (same donor as pbmc8k). PBMCs are primary cells with relatively small amounts of RNA (1pg RNA/cell). It is downloaded from https://www.10xgenomics.com/.

S3 Results

S3.1 State-of-art Methods for Comparison

In this article, nine state-of-art imputation methods are used for comparison, namely scImpute[14], DrImpute[15], scDoc[16], scRMD[17], ALRA[18], MAGIC[19], SAVER[20], DeepImpute[21]and scVI [22]. The availability of these methods is as follows:

- scImpute: The R package of scImpute can be downloaded from https://github.com/Vivianstats/scImpute and it is implemented with default parameters.
- DrImpute: We download the R package of DrImpute from https://github.com/gongx030/DrImpute and implement it with default parameters.
- scDoc: The R package of scDoc can be downloaded from https://github.com/anlingUA/scDoc and it is implemented with default parameters.
- scRMD: The R package of scRMD is downloaded from https://github.com/XiDsLab/scRMD, and we implement it with default parameters.
- ALRA: The R source codes of ALRA can be aquired from https://github.com/KlugerLab/ALRA, we run the source codes with default parameters.
- MAGIC: The R package of MAGIC is aquired from https://github.com/KrishnaswamyLab/MAGIC, and the algorithm is implemented with deault parameters.
- SAVER: The R package of SAVER can be downloaed from https://github.com/mohuangx/SAVER and it is implemented with default parameters.
- DeepImpute: The Python package of DeepImpute can be downloaed from https://github.com/lanagarmire/deepimpute and it is implemented with default parameters.
- scVI: The Python package of scVI can be downloaded from https://github.com/scverse/scvi-tools, and the algorithm is implemented with deault parameters.

S3.2 Evaluation metrics

In this study, we employ several evaluation metrics to assess the performance of different imputation methods. The formulations of these metrics are presented in this section and include the Root Mean Squared Error (RMSE), Mean Absolute Error (MAE), Spearman correlation coefficient, Pearson correlation coefficient, sensitivity, Adjusted Rand index (ARI)[23], and Normalized Mutual Information (NMI)[24].

RMSE and MAE

Let $X = (x_1, x_2, \ldots, x_n)$ denote the vector of true values, and $\hat{X} = (\hat{x}_1, \hat{x}_2, \ldots, \hat{x}_n)$ denote the vector of predicted values. The *Root Mean Squared Error (RMSE)* and *Mean Absolute Error (MAE)* are defined as:

$$\text{RMSE} = \sqrt{\frac{\sum_{i=1}^{n}(x_i - \hat{x}_i)^2}{n}} \tag{3}$$

$$\text{MAE} = \frac{\sum_{i=1}^{n}|x_i - \hat{x}_i|}{n} \tag{4}$$

Pearson and Spearman Correlation Coefficients

Let $X = (x_1, x_2, \ldots, x_n)$ denote the true values and $Y = (y_1, y_2, \ldots, y_n)$ denote the predicted values. The *Pearson correlation coefficient* is given by:

$$\rho_p = \frac{\sum_{i=1}^{n}(x_i - \bar{x})(y_i - \bar{y})}{\sqrt{\sum_{i=1}^{n}(x_i - \bar{x})^2}\sqrt{\sum_{i=1}^{n}(y_i - \bar{y})^2}} \tag{5}$$

where $\bar{x}$ and $\bar{y}$ are the means of X and Y, respectively.

The *Spearman rank correlation coefficient* is defined as:

$$\rho_s = 1 - \frac{6\sum_{i=1}^{n} d_i^2}{n(n^2 - 1)} \tag{6}$$

where d_i denotes the difference in ranks between x_i and y_i.

ARI and NMI

Adjusted Rand Index (ARI) and Normalized Mutual Information (NMI) are two popular evaluation metrics for assessing clustering performance. The ARI is defined as:

$$\mathrm{ARI}(A^*, A) = \frac{\sum_{i,j}\binom{N_{ij}}{2} - \frac{\sum_i \binom{N_{i\cdot}}{2}\sum_j \binom{N_{\cdot j}}{2}}{\binom{N}{2}}}{\frac{1}{2}\left[\sum_i \binom{N_{i\cdot}}{2} + \sum_j \binom{N_{\cdot j}}{2}\right] - \frac{\sum_i \binom{N_{i\cdot}}{2}\sum_j \binom{N_{\cdot j}}{2}}{\binom{N}{2}}}, \tag{6}$$

where:

- N is the total number of cells.
- N_{ij} is the number of cells of real cell type $C_j^* \in A^*$ assigned to cluster C_i in partition A.
- $N_{i\cdot}$ is the number of cells in cluster C_i.
- $N_{\cdot j}$ is the number of cells of cell type C_j^*.

The ARI ranges from -1 to 1, where higher values indicate better clustering quality. An ARI of 1 implies that the clustering result is identical to the ground truth partition.

The NMI is defined as:

$$\mathrm{NMI}(A^*, A) = \frac{2 \times I(A^*, A)}{H(A^*) + H(A)}, \tag{7}$$

where:

$$I(A^*, A) = H(A^*) - H(A^* \mid A), \tag{8}$$

$$H(A) = -\sum_{a \in A} p(a)\log_2 p(a), \tag{9}$$

$$H(A^* \mid A) = H(A^*, A) - H(A), \tag{10}$$

$$H(A^*, A) = -\sum_{a^* \in A^*}\sum_{a \in A} p(a^*, a)\log_2 p(a^*, a). \tag{11}$$

The NMI ranges from 0 to 1, with larger values indicating better clustering performance.

Precision, Recall, and F1-score

Precision, Recall, and F1-score are commonly used metrics for evaluating the accuracy of classification or detection tasks. In the context of dropout identification, these metrics can be defined as follows:

Precision measures the proportion of correctly identified dropout events among all events predicted as dropouts:

$$\text{Precision} = \frac{\text{TP}}{\text{TP} + \text{FP}}, \tag{12}$$

where TP denotes the number of true positives and FP denotes the number of false positives.

Recall measures the proportion of correctly identified dropout events among all actual dropouts:

$$\text{Recall} = \frac{\text{TP}}{\text{TP} + \text{FN}}, \tag{13}$$

where FN denotes the number of false negatives.

F1-score is the harmonic mean of Precision and Recall, providing a balanced measure between them:

$$\text{F1-score} = \frac{2 \times \text{Precision} \times \text{Recall}}{\text{Precision} + \text{Recall}}. \tag{14}$$

The values of Precision, Recall, and F1-score range between 0 and 1, with higher values indicating better performance in identifying dropouts.

S4 Supplementary tables and figures

Table S1 Pearson correlation across different dropout levels for all imputation methods.

Method	30%	40%	50%	60%	70%	80%
scImpute	0.968	0.956	0.939	0.910	0.880	0.720
DrImpute	0.965	0.942	0.905	0.855	0.785	0.700
scDoc	0.930	0.890	0.830	0.760	0.670	0.550
scRMD	0.980	0.957	0.937	0.910	0.885	0.570
ALRA	0.915	0.857	0.843	0.640	0.610	0.480
MAGIC	0.962	0.954	0.946	0.900	0.890	0.730
SAVER	0.925	0.900	0.850	0.790	0.710	0.590
DeepImpute	0.964	0.943	0.920	0.880	0.830	0.730
scVI	0.856	0.835	0.820	0.780	0.750	0.710
scDDI	0.979	0.964	0.940	0.900	0.840	0.720

Table S2 Spearman rank correlation across different dropout levels for all imputation methods.

Method	30%	40%	50%	60%	70%	80%
scImpute	0.915	0.900	0.880	0.860	0.830	0.790
DrImpute	0.920	0.910	0.890	0.870	0.840	0.810
scDoc	0.775	0.710	0.630	0.530	0.480	0.430
scRMD	0.735	0.650	0.570	0.500	0.420	0.380
ALRA	0.798	0.790	0.768	0.510	0.499	0.410
MAGIC	0.860	0.860	0.858	0.856	0.850	0.800
SAVER	0.830	0.780	0.750	0.690	0.610	0.530
DeepImpute	0.840	0.850	0.840	0.830	0.820	0.810
scVI	0.760	0.770	0.780	0.780	0.776	0.758
scDDI	0.910	0.910	0.880	0.830	0.790	0.720

Table S3 RMSE values across five masked datasets (5% masking) for all imputation methods.

Method	Darmanis	Goolam	Pollen	Yan	Ting
scImpute	3.89	2.39	4.03	1.65	4.87
DrImpute	1.93	1.45	2.17	1.23	3.78
scDoc	2.92	2.53	3.24	1.59	3.69
scRMD	2.24	1.30	2.26	1.06	3.01
ALRA	1.69	2.04	1.79	1.11	2.84
MAGIC	1.93	1.24	2.16	1.03	3.89
SAVER	2.62	2.95	2.77	1.53	4.74
DeepImpute	1.37	3.84	1.63	2.64	4.89
scVI	2.26	4.65	3.73	2.88	4.79
scDDI	1.39	1.21	1.92	1.03	3.40

Table S4 MAE values across five masked datasets (5% masking) for all imputation methods.

Method	Darmanis	Goolam	Pollen	Yan	Ting
scImpute	2.80	1.74	3.07	1.24	3.87
DrImpute	1.58	1.55	1.76	0.99	3.76
scDoc	2.31	2.97	2.65	1.14	3.25
scRMD	1.84	1.01	1.80	0.85	2.79
ALRA	1.33	1.36	1.34	0.80	2.51
MAGIC	1.57	0.98	1.75	0.81	3.13
SAVER	2.30	1.62	2.53	1.92	2.81
DeepImpute	1.11	2.82	1.28	1.97	2.79
scVI	1.84	4.31	3.48	2.65	2.85
scDDI	1.30	0.96	1.49	0.80	2.81

Table S5 Results of ablation study of scDDI on six real datasets measured by NMI

PNB	WCS	Regressor	Darmanis	Goolam	Pollen	Yan	Ting	PBMC
✓	✓	✓	**0.51**	**0.79**	**0.88**	**0.87**	**0.76**	**0.61**
✓	✓	✗	0.48	0.64	0.82	0.87	0.64	0.59
✓	✗	✓	0.49	0.77	0.85	0.83	0.63	0.58
✓	✗	✗	0.50	0.64	0.85	0.84	0.61	0.56
✗	✓	✓	0.50	0.78	0.51	0.83	0.63	0.58
✗	✓	✗	0.46	0.66	0.83	0.82	0.65	0.55
✗	✗	✓	0.49	0.77	0.85	0.83	0.64	0.53
✗	✗	✗	0.48	0.66	0.85	0.82	0.62	0.52

Note. ✓ indicates that the component is included; ✗ indicates that it is removed or replaced. NMI = Normalized Mutual Information. The full model (✓ ✓ ✓; i.e., **PNB–WCS–Rrgressor**) consistently achieves the highest clustering performance across all datasets.

Table S6 ARI results (Mean ± 95% CI) and Wilcoxon signed-rank test p-values vs scDDI for simulated datasets

Method	ARI Mean ± CI	p-value vs scDDI
scDDI	0.549 ± 0.164	–
scImpute	0.499 ± 0.199	0.0469
DrImpute	0.485 ± 0.162	0.0312
scDoc	0.497 ± 0.174	0.0312
scRMD	0.487 ± 0.205	0.0938
ALRA	0.482 ± 0.194	0.1562
MAGIC	0.417 ± 0.231	0.0781
SAVER	0.491 ± 0.192	0.0469
DeepImpute	0.479 ± 0.185	0.0781
scVI	0.444 ± 0.130	0.0312

Table S7 NMI results (Mean ± 95% CI) and Wilcoxon signed-rank test p-values vs scDDI for simulated datasets

Method	NMI Mean ± CI	p-value vs scDDI
scDDI	0.733 ± 0.127	–
scImpute	0.689 ± 0.144	0.0312
DrImpute	0.693 ± 0.116	0.0312
scDoc	0.689 ± 0.132	0.0781
scRMD	0.691 ± 0.141	0.0156
ALRA	0.685 ± 0.133	0.0781
MAGIC	0.647 ± 0.156	0.0312
SAVER	0.699 ± 0.128	0.0156
DeepImpute	0.663 ± 0.179	0.0469
scVI	0.661 ± 0.117	0.0312

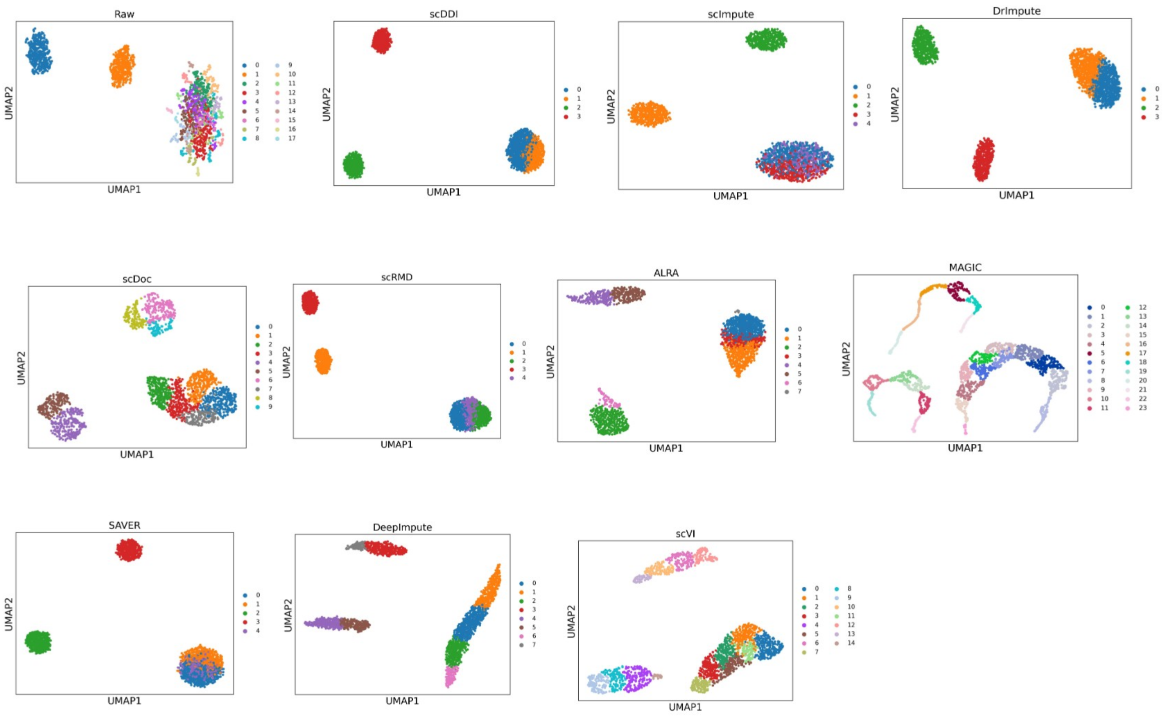

Fig S1 Comparison of clustering results on the imputed Simulated Dataset 7 across eleven methods: the raw data, scDDI, and nine other state-of-the-art approaches.

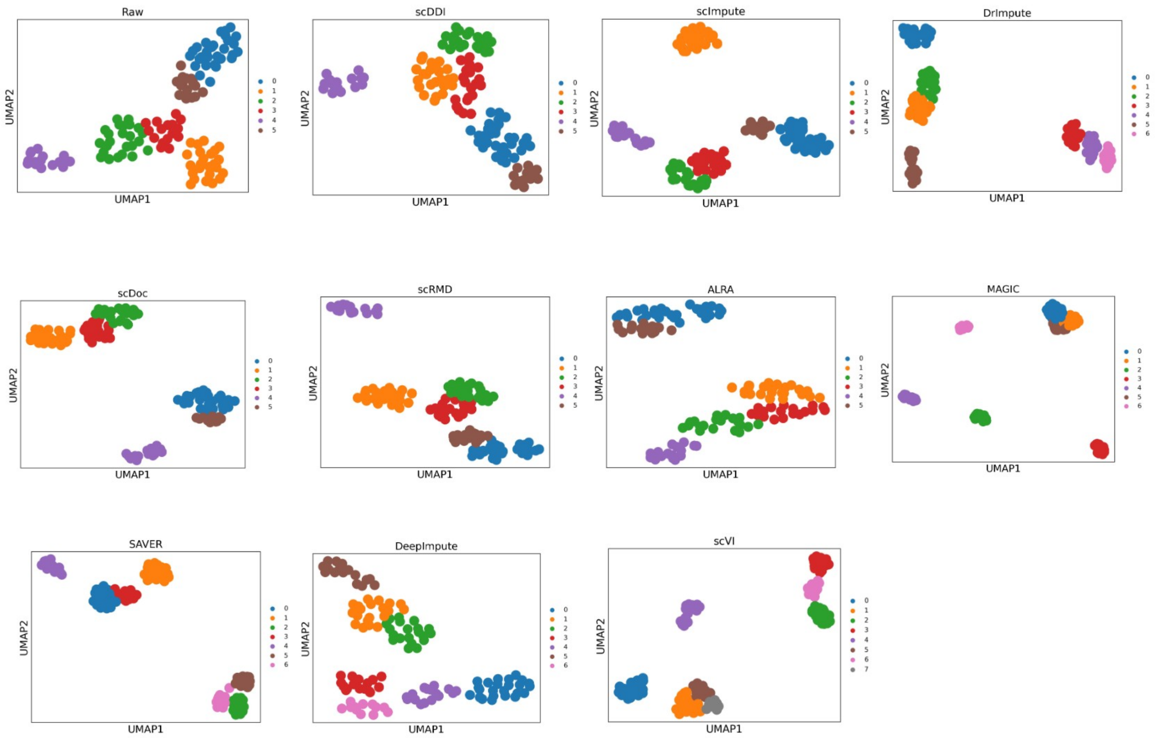

Fig S2 Comparison of clustering results on the imputed Goolam dataset across eleven methods: the raw data, scDDI, and nine other state-of-the-art approaches.

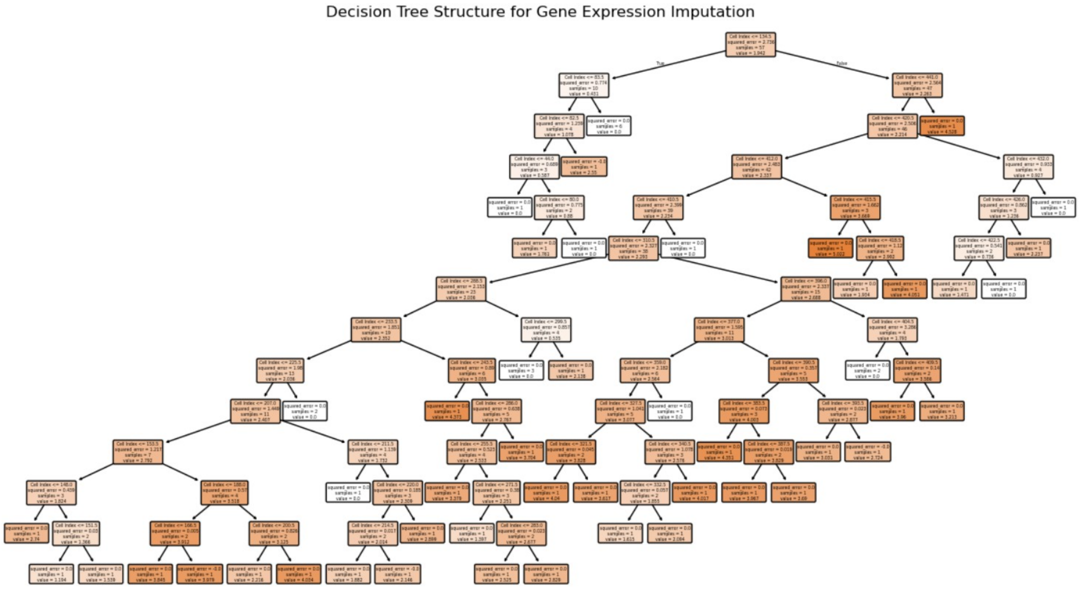

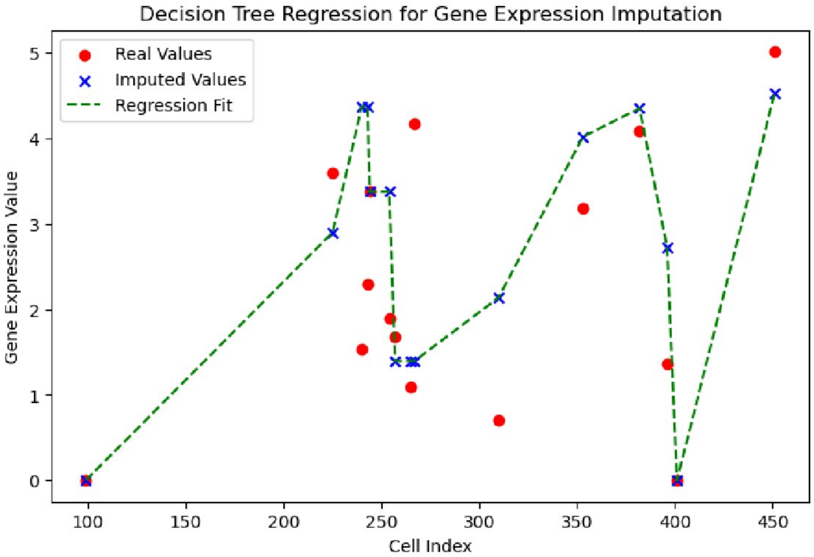

Fig S3 Figure shows Decision Tree Regression for imputation of Darmanis dataset.

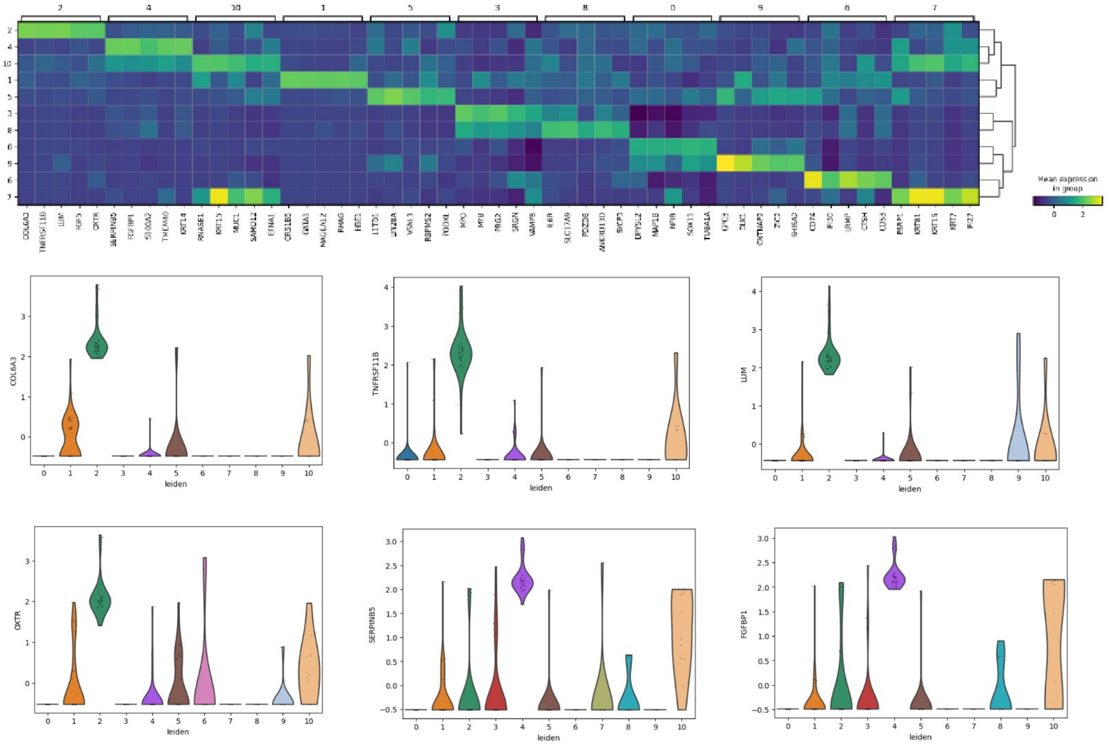

Fig S4 Figure shows marker gene analysis for imputed Pollen dataset. The average expression values of the top five DE genes are shown in heatmap. The violin plots of the expression profiles of those top DE genes within each cluster are also shown.

References

[1] Shun H. Yip, Panwen Wang, Jean-Pierre A. Kocher, et al. Linnorm: improved statistical analysis for single cell RNA-seq expression data. *Nucleic Acids Research*, 45(22):e179–e179, 09 2017.

[2] Yuhan Hao, Tim Stuart, Madeline H. Kowalski, et al. Dictionary learning for integrative, multimodal and scalable single-cell analysis. *Nature Biotechnology*, 42(2):293–304, Feb 2024.

[3] Koen Van den Berge, Fanny Perraudeau, Charlotte Soneson, et al. Observation weights unlock bulk rna-seq tools for zero inflation and single-cell applications. *Genome Biology*, 19(1):24, Feb 2018.

[4] A. P. Dempster, N. M. Laird, and D. B. Rubin. Maximum likelihood from incomplete data via the em algorithm. *Journal of the Royal Statistical Society: Series B (Methodological)*, 39(1):1–22, 12 2018.

[5] Jitendra Singh Kushwah, Atul Kumar, Subhash Patel, et al. Comparative study of regressor and classifier with decision tree using modern tools. *Materials Today: Proceedings*, 56:3571–3576, 2022. First International Conference on Design and Materials.

[6] F. Pedregosa, G. Varoquaux, A. Gramfort, and others. Scikit-learn: Machine learning in Python. *Journal of Machine Learning Research*, 12:2825–2830, 2011.

[7] Liying Yan, Mingyu Yang, Hongshan Guo, et al. Single-cell rna-seq profiling of human preimplantation embryos and embryonic stem cells. *Nature Structural & Molecular Biology*, 20(9):1131–1139, Sep 2013.

[8] David T Ting, Ben S Wittner, Matteo Ligorio, et al. Single-cell RNA sequencing identifies extracellular matrix gene expression by pancreatic circulating tumor cells. *Cell Rep*, 8(6):1905–1918, September 2014.

[9] Mubeen Goolam, Antonio Scialdone, Sarah JL Graham, et al. Heterogeneity in oct4 and sox2 targets biases cell fate in 4-cell mouse embryos. *Cell*, 165(1):61–74, 2016.

[10] Alex A Pollen, Tomasz J Nowakowski, Joe Shuga, et al. Low-coverage single-cell mrna sequencing reveals cellular heterogeneity and activated signaling pathways in developing cerebral cortex. *Nature biotechnology*, 32(10):1053, 2014.

[11] Spyros Darmanis, Steven A Sloan, Ye Zhang, et al. A survey of human brain transcriptome diversity at the single cell level. *Proceedings of the National Academy of Sciences*, 112(23):7285–7290, 2015.

[12] Itay Tirosh, Benjamin Izar, Sanjay M Prakadan, et al. Dissecting the multicellular ecosystem of metastatic melanoma by single-cell rna-seq. *Science*, 352(6282):189–196, 2016.

[13] Grace X. Y. Zheng, Jessica M. Terry, Phillip Belgrader, et al. Massively parallel digital transcriptional profiling of single cells. *Nature Communications*, 8(1):14049, Jan 2017.

[14] Wei Vivian Li and Jingyi Jessica Li. An accurate and robust imputation method scimpute for single-cell rna-seq data. *Nature Communications*, 9(1):997, Mar 2018.

[15] Wuming Gong, Il-Youp Kwak, Pruthvi Pota, et al. Drimpute: imputing dropout events in single cell rna sequencing data. *BMC Bioinformatics*, 19(1):220, Jun 2018.

[16] Di Ran, Shanshan Zhang, Nicholas Lytal, et al. scDoc: correcting drop-out events in single-cell RNA-seq data. *Bioinformatics*, 36(15):4233–4239, 05 2020.

[17] Chong Chen, Changjing Wu, Linjie Wu, et al. scRMD: imputation for single cell RNA-seq data via robust matrix decomposition. *Bioinformatics*, 36(10):3156–3161, 03 2020.

[18] George C. Linderman, Jun Zhao, Manolis Roulis, et al. Zero-preserving imputation of single-cell rna-seq data. *Nature Communications*, 13(1):192, Jan 2022.

[19] David van Dijk, Roshan Sharma, Juozas Nainys, et al. Recovering gene interactions from single-cell data using data diffusion. *Cell*, 174(3):716–729.e27, Jul 2018.

[20] Mo Huang, Jingshu Wang, Eduardo Torre, et al. Saver: gene expression recovery for single-cell rna sequencing. *Nature Methods*, 15(7):539–542, Jul 2018.

[21] Cédric Arisdakessian, Olivier Poirion, Breck Yunits, et al. Deepimpute: an accurate, fast, and scalable deep neural network method to impute single-cell rna-seq data. *Genome Biology*, 20(1):211, Oct 2019.

[22] Romain Lopez, Jeffrey Regier, Michael B. Cole, et al. Deep generative modeling for single-cell transcriptomics. *Nature Methods*, 15(12):1053–1058, Dec 2018.

[23] Lawrence Hubert and Phipps Arabie. Comparing partitions. *Journal of Classification*, 2(1):193–218, Dec 1985.

[24] Alexander Strehl and Joydeep Ghosh. Cluster ensembles – a knowledge reuse framework for combining multiple partitions. *Journal of Machine Learning Research*, 3:583–617, 2002.
